# Supplementary material for: Uncovering Plant Virus Species Forming Novel Provisional Taxonomic Units Related to the Family Benyviridae
Source: Viruses. 2022 Nov 29;14(12):2680. doi: 10.3390/v14122680 (PMC9781952; doi:10.3390/v14122680)
Supplement: Supplementary file 1 [file viruses-14-02680-s001.zip › viruses-1953767-supplementary.pdf]

Supplementary Figures

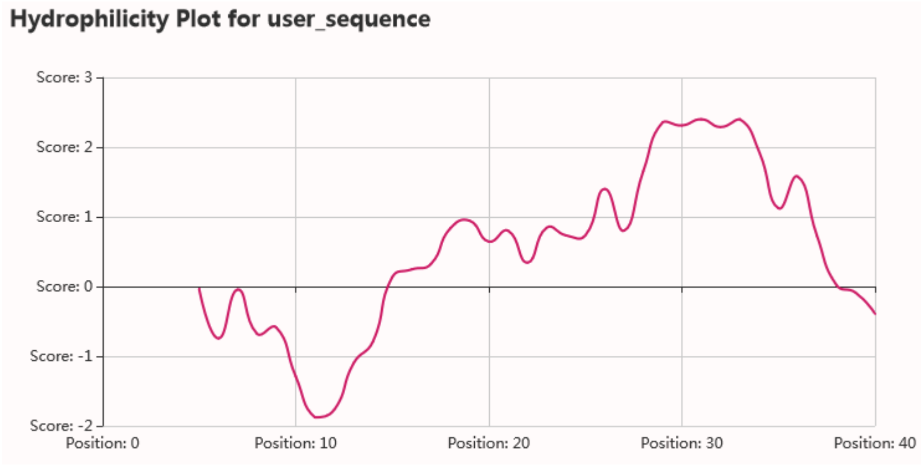

Figure S1. Prediction of potential hydrophobic membrane-bound regions in ORF4 protein of *Diplonema papillatum* VLRA.

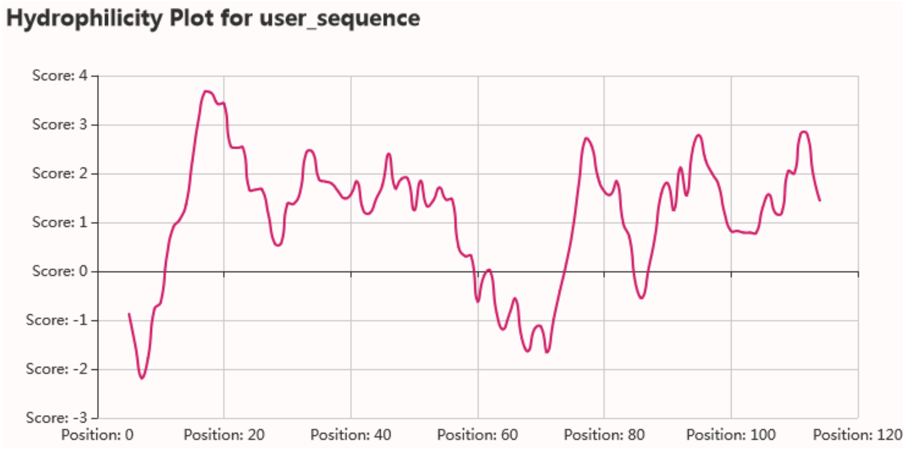

Figure S2. Prediction of potential hydrophobic membrane-bound regions in *Salvia miltiorrhiza* VLRA ORF3 protein.

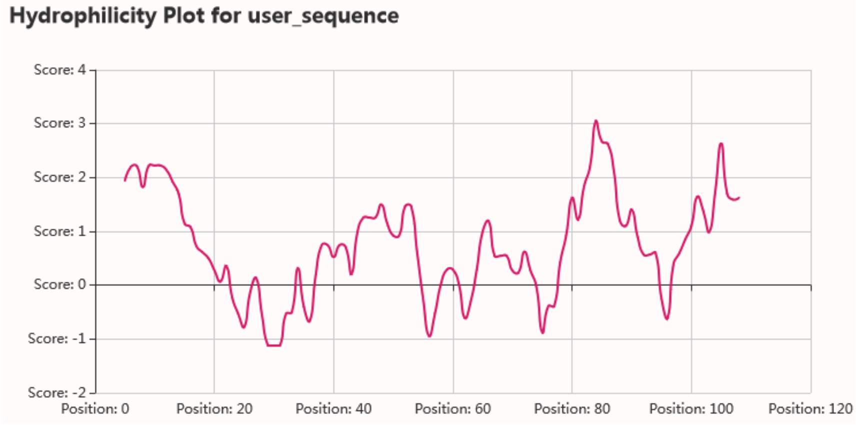

Figure S3. Prediction of potential hydrophobic membrane-bound regions in *Red clover RNA virus 1* VLRA ORF2 protein.

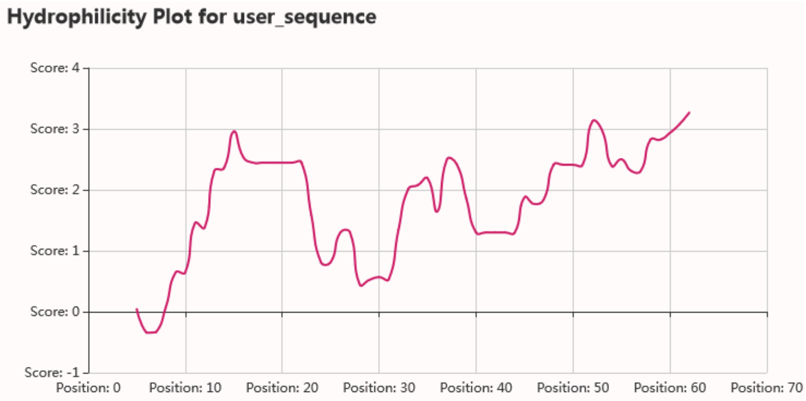

Figure S4. Prediction of potential hydrophobic membrane-bound regions in *Red clover RNA virus 1* VLRA ORF3 protein.

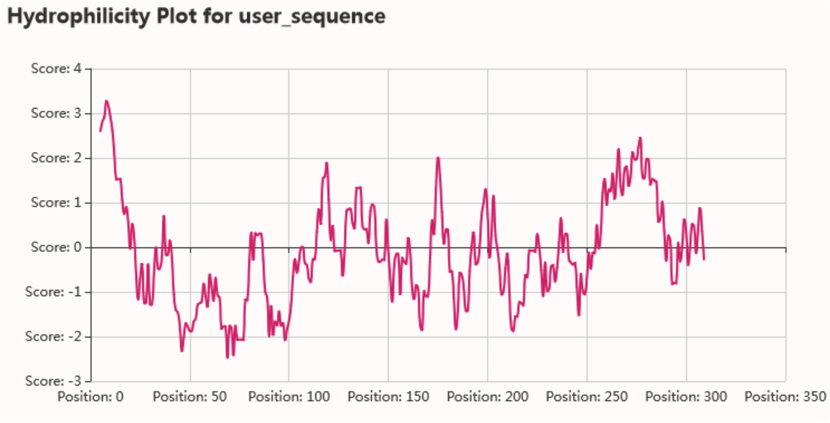

Figure S5. Prediction of potential hydrophobic membrane-bound regions in *Rhyncholacis penicillata* Rhyc27837 VLRA ORF2 protein.

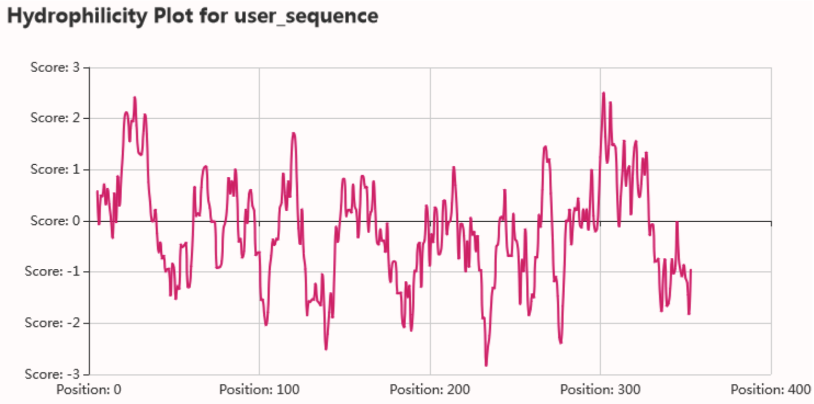

Figure S6. Prediction of potential hydrophobic membrane-bound regions in *Rhyncholacis penicillata* Rhyc16 VLRA ORF2 protein.

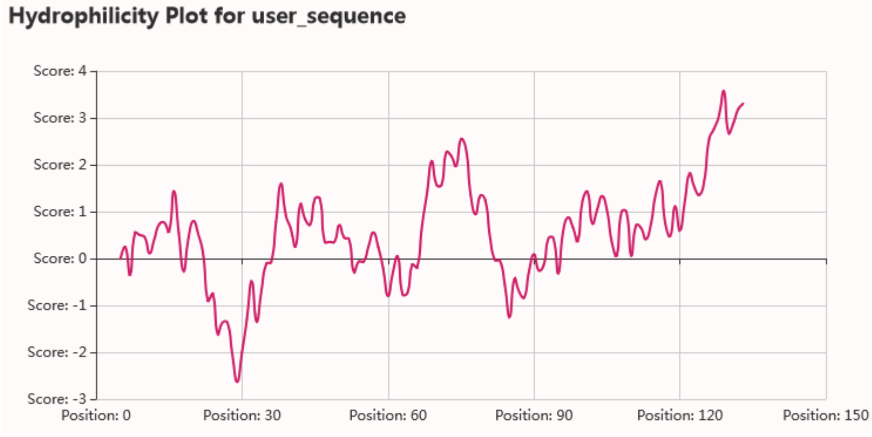

Figure S7. Prediction of potential hydrophobic membrane-bound regions in ORF2 protein of *Dactylorhiza hatagirea beny-like virus*.

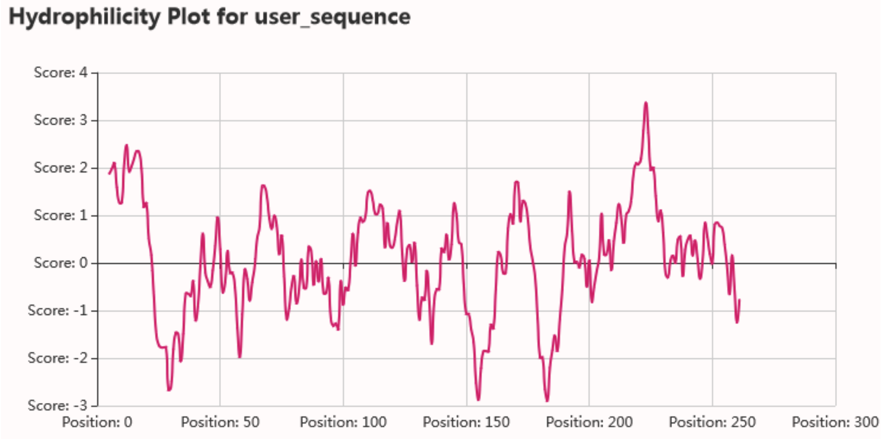

Figure S8. Prediction of potential hydrophobic membrane-bound regions in ORF2 proteins of *Arceuthobium sichuanense virus 3*.

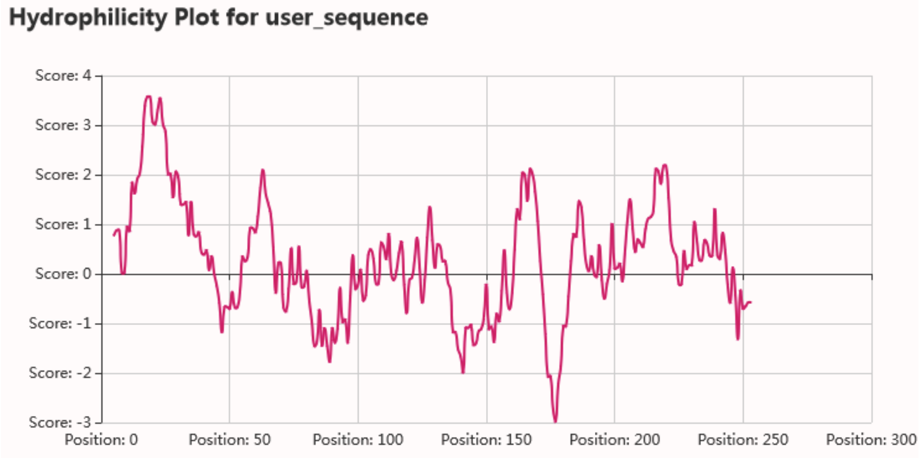

Figure S9. Prediction of potential hydrophobic membrane-bound regions in ORF2 proteins of *Viscum album* VLRA.

Supplementary Tables

Table S1. Pairwise sequence comparisons of *Agarophyton vermiculophyllum* VLRA-encoded HEL domain with selected viral helicase domains of other viruses and VLRAs.

Subject virus or VLRA source*	E-value	Amino acid sequence identity (%)	NCBI accession
VLRA from *Quercus castanea*	9e-32	42	GHJU01198988
VLRA from *Rhyncholacis cf. penicillata*	7e-28	40	ICSC01056734
VLRA from *Silene dioica*	3e-28	39	GFCG01071918
VLRA from *Lithophyllum crustose*	2e-24	38	GHIV01061204
Red clover RNA virus 1	1e-26	40	MG596242
Arceuthobium sichuanense virus 3	2e-27	39	BK059270
Sanya benyvirus 1	7e-28	39	MZ209861
Hubei Beny-like virus 1	2e-28	39	OL700057
Bemisia tabaci beny-like virus 6	2e-26	38	MW256699

* Viruses with plant hosts are shown in green; red algae hosts – in pink; arthropod hosts – in blue.

Table S2. Pairwise sequence comparisons of *Lithophyllum crustose* coralline algae DN227823 VLRA-encoded HEL domain with selected viral helicase domains of other viruses and VLRAs.

Subject virus or VLRA source*	E-value	Amino acid sequence identity (%)	NCBI accession
Sanya benyvirus 1	4e-56	52	UHK03121
Diabrotica undecimpunctata virus 2	3e-52	51	QIT20101
Sichuan mosquito Beny-like virus	5e-49	50	UBJ25988
Guiyang benyvirus 1	3e-51	49	UHK03084
Hubei Beny-like virus 1	1e-49	48	OL700057
Bemisia tabaci beny-like virus 6	7e-49	48	MW256699
Pistacia ribo-like virus	7e-45	47	QPL17790
Goji berry chlorosis virus	6e-34	42	AYO99569
VLRA from *Quercus castanea*	1e-31	41	GHJU01198988
Rice stripe necrosis virus	1e-33	38	QHN70741
VLRA from *Agarophyton vermiculophyllum*	2e-24	38	GILD01050008

* Viruses with plant hosts are shown in green; red algae hosts – in pink; arthropod hosts – in blue.

Table S3. Pairwise sequence comparisons of *Shorea curtisii* VLRA-encoded HEL domain of ORF2 (BMB1) protein with selected viral helicase domains of other viruses and VLRAs.

Subject virus or VLRA source*	E-value	Amino acid sequence identity (%)	NCBI accession
Quercus castanea BMB1	3e-35	44	GHJU01198988
Litchi chinensis BMB1	2e-30	39	GAIP01007090
Hibiscus green spot virus 2 BMB1	6e-25	38	YP_004928123
Lathyrus sativus BMB1	4e-28	35	GBSS01016353
Pistachio virus X BMB1	2e-17	31	QPL17813
Shorea curtisii POL	2e-12	33	GJMJ01032282
Quercus castanea POL	7e-12	30	GHJU01198988
Colobanthus quitensis TCMB1	5e-17	32	GCIB01126289

* Binamovirids are shown in green; Higreviruses – in blue; Tecimovirids – in yellow.
